# Oligonucleotide Therapy for Obstructive and Restrictive Respiratory Diseases

**DOI:** 10.3390/molecules22010139

**Published:** 2017-01-17

**Authors:** Wupeng Liao, Jinrui Dong, Hong Yong Peh, Lay Hong Tan, Kah Suan Lim, Li Li, Wai-Shiu Fred Wong

**Affiliations:** 1 Department of Pharmacology, Yong Loo Lin School of Medicine, National University Health System, Singapore 117600, Singapore; phcliao@nus.edu.sg (W.L.); a0109375@u.nus.edu (J.D.); pehhongyong@u.nus.edu (H.Y.P.); invisible_bubbles@hotmail.com (L.H.T.); kahsuan339@gmail.com (K.S.L.); lilinku2010@163.com (L.L.); 2 Immunology Program, Life Science Institute, National University of Singapore, Singapore 117456, Singapore; 3 CREATE Program, National University of Singapore, Singapore 138602, Singapore

**Keywords:** asthma, COPD, pulmonary fibrosis, antisense oligonucleotide, small-interfering RNA, microRNA

## Abstract

Inhaled oligonucleotide is an emerging therapeutic modality for various common respiratory diseases, including obstructive airway diseases like asthma and chronic obstructive pulmonary disease (COPD) and restrictive airway diseases like idiopathic pulmonary fibrosis (IPF). The advantage of direct accessibility for oligonucleotide molecules to the lung target sites, bypassing systemic administration, makes this therapeutic approach promising with minimized potential systemic side effects. Asthma, COPD, and IPF are common chronic respiratory diseases, characterized by persistent airway inflammation and dysregulated tissue repair and remodeling, although each individual disease has its unique etiology. Corticosteroids have been widely prescribed for the treatment of asthma, COPD, and IPF. However, the effectiveness of corticosteroids as an anti-inflammatory drug is limited by steroid resistance in severe asthma, the majority of COPD cases, and pulmonary fibrosis. There is an urgent medical need to develop target-specific drugs for the treatment of these respiratory conditions. Oligonucleotide therapies, including antisense oligonucleotide (ASO), small interfering RNA (siRNA), and microRNA (miRNA) are now being evaluated both pre-clinically and clinically as potential therapeutics. The mechanisms of action of ASO and siRNA are highly target mRNA specific, ultimately leading to target protein knockdown. miRNA has both biomarker and therapeutic values, and its knockdown by a miRNA antagonist (antagomir) has a broader but potentially more non-specific biological outcome. This review will compile the current findings of oligonucleotide therapeutic targets, verified in various respiratory disease models and in clinical trials, and evaluate different chemical modification approaches to improve the stability and potency of oligonucleotides for the treatment of respiratory diseases.

## 1. Major Respiratory Diseases and the Current Therapies

### 1.1. Asthma

There are more than 300 million people who suffer from asthma, with an estimated death rate of approximately 250,000 patients annually [1,2]. In 2007, US$56 billion in healthcare costs was incurred in the US alone and about €18 billion in Europe [3,4]. The classic hallmarks of this obstructive airway disease are airway inflammation, airway remodeling, mucus hypersecretion, and airway hyperresponsiveness (AHR) [5]. The hallmarks can be attributed to leukocyte infiltration in the lungs, including lymphocytes, eosinophils and neutrophils, increased production of pro-inflammatory cytokines (IL-4, IL-5, IL-13, GM-CSF and IL-17), and elevated lgE level [5,6].

Current treatments of asthma include inhaled corticosteroids (ICS) to suppress lung inflammation and β_2_ agonists as bronchodilators [5]. While such standard therapies are able to relieve asthma symptoms in most patients, there are approximately 10% of asthmatics who are diagnosed with severe asthma that are less responsive or resistant to ICS [7]. Alternative drug treatments such as leukotriene modifiers, mast cell stabilizers, and anti-lgE antibodies are also not capable of controlling asthma symptoms in severe asthmatics [5]. There is an unmet medical need to discover a cure for asthma and treat severe asthmatics that are resistant to corticosteroid therapy.

### 1.2. Chronic Obstructive Pulmonary Disease (COPD)

According to the World Health Organization, approximately 65 million people suffer from moderate to severe COPD. There were 3 million deaths from COPD in 2005, which corresponds to 5% of all deaths in that year [8]. In 2010, COPD, another major obstructive airway disease, was the fourth leading cause of death, and is projected to be the third leading cause of death by 2020 [9]. The healthcare cost for COPD in 2010 was estimated at US$50 billion in the US and US$2.1 trillion globally [10,11]. Common symptoms of COPD include chronic coughing, difficulty breathing, and emphysema, mainly due to airway inflammation, peribronchiolar fibrosis, and the destruction of alveolar sacs [12]. The major risk factor for COPD is cigarette smoking, which contains massive amounts of reactive oxidants [13]. In bronchoalveolar lavage (BAL) fluid from COPD patients, increased numbers of inflammatory cells (neutrophils and macrophages), coupled with elevated levels of pro-inflammatory cytokines/chemokines and matrix metalloproteinases, have been observed [14].

Besides implementing preventative steps for the cessation of smoking, there are currently no effective treatments to control or delay the disease progression of COPD. Notably, ICS treatments are less effective in COPD than in asthma, as more patients are resistant to corticosteroids [15]. Roflumilast, a selective phosphodiesterase (PDE) 4 inhibitor specially indicated for COPD treatment, unfortunately has a low therapeutic index in COPD with dose-limiting major side effects [16]. Therefore, it is urgent to discover novel therapeutic agents for COPD treatment.

### 1.3. Pulmonary Fibrosis

Major pulmonary fibrosis diseases include cystic fibrosis and idiopathic pulmonary fibrosis (IPF). IPF is characterized by a chronic progressive decline in lung function, which is fatal at the end-stage. The scarring of lung tissue causes worsening dyspnea (shortness of breath) in IPF. The prevalence of IPF was estimated to be 63 per 100,000 persons in the US [17]. IPF is a restrictive airway disease and proposed to be the consequence of an aberrant tissue repair process, which results in inflammation and excessive collagen deposition in lung interstitium [18]. High levels of transforming growth factor (TGF)-β stimulate and differentiate airway fibroblasts into myofibroblasts, which are particularly active in extracellular matrix deposition, contributing to pulmonary fibrosis and airway remodeling [18].

Current treatments of pulmonary fibrosis aim at relieving symptoms and preventing acute exacerbations, by reducing mucus secretion and attenuating bacterial infection [19]. Current pharmacological interventions to IPF include pirfenidone (a small molecule that possesses anti-inflammatory, anti-oxidative, and anti-fibrotic effects) [20], *N*-acetylcystein (an antioxidant), and receptor tyrosine kinase inhibitor nintedanib [21]. Newer therapies can be designed to target essential growth factors and cytokines responsible for fibroblast proliferation, activation, transformation, and survival.

## 2. Oligonucleotides as Therapeutics for Respiratory Diseases

Oligonucleotide therapy has great potential for the treatment of human diseases, with FDA approval of antisense drugs like Fomivirsen [22] for the treatment of cytomegalovirus retinitis; and Kynamro for the treatment of homozygous familial hypercholesterolemia [23]; and with on-going clinical trials, in using small interfering RNA (siRNA) [24] and splice switching oligonucleotides [25]. The entry of oligonucleotide into cells depends on the structure and concentration of the oligonucleotide and the cell types. Adsorptive endocytosis and fluid phase pinocytosis are believed to be the major mechanisms of oligonucleotide internalization. At a relatively low concentration, the internalization of oligonucleotide occurs via interaction with membrane-bound receptors in a process called adsorptive endocytosis, whereas, at a relatively high concentration, pinocytosis plays a major role in oligonucleotide internalization due to the saturation of membrane-bound receptors [26]. However, effective delivery of the oligonucleotides to their intracellular sites of action still remains a major challenge. Current approaches to facilitate oligonucleotide delivery include lipid- and polymer-based nanoparticles and ligand-oligonucleotide conjugates such as lipid, peptide, antibody, and aptamer conjugates, and so on [27]. While the majority of oligonucleotide therapies are given systemically, the respiratory track provides an ideal site for topical delivery for respiratory diseases. The airways are uniquely lined with pulmonary surfactants, which are primarily composed of zwitterionic lipids. These surfactant lipids possess cationic properties at the pH of the respiratory tract. When anionic oligonucleotides are inhaled, they tend to be adsorbed by the surfactants, resulting in reformulated particles that have been hypothesized to be efficiently taken up into the cells [28]. This review mainly focuses on the discovery and development of target-specific oligonucleotides that have been validated in pre-clinical animal models or tested in clinical trials for the treatment of major obstructive and restrictive respiratory diseases, including asthma, COPD, and pulmonary fibrosis.

### 2.1. Antisense Oligonucleotide (ASO)

ASO is a single-stranded DNA or RNA (typically 20–21 bp in length) with a sequence that binds complementarily to the target mRNA through Watson-Crick base pairing, resulting in target gene knockdown [29]. Since the first observation of ASO-mediated gene knockdown of the Rous sarcoma viral RNA, a great number of studies have been conducted to explore the therapeutic application of ASO in silencing dysregulated genes in a variety of human diseases [30]. Two major classes of mechanism are proposed to induce loss-of-function of a particular gene; activation of RNase H enzyme and steric blockade of the ribosome complex.

**Figure 1:**
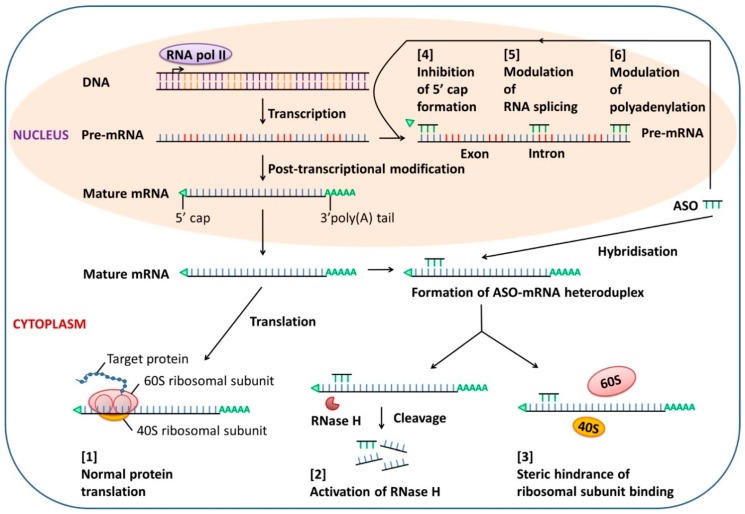
Mechanisms of action of antisense oligonucleotide (ASO). In the absence of ASO, normal gene transcription and protein translation are maintained [1]. ASO can enter cells by endocytosis and hybridize with target mRNA in the cytoplasm. Formation of an ASO-mRNA heteroduplex activates RNase H, leading to the degradation of target mRNA [2], or interferes with ribosomal assembly by steric hindrance, resulting in inhibition of translation [3]. Both actions will knock down the target protein. In addition, the binding of ASO to the target pre-mRNA in the nucleus can regulate mRNA maturation through [4] inhibition of 5′ cap formation, [5] modulation of RNA splicing, and [6] blockade of polyadenylation.

Most ASOs act through RNase H-mediated cleavage of the ASO-targeted mRNA, leading to the degradation of the target mRNA, leaving the ASO intact for re-use. Some ASOs target the 5′ end or AUG initiation codon region of the target mRNA to prevent translation by steric hindrance of ribosomal activity [31]. Other mechanisms of ASO-mediated gene silencing include interference with mRNA maturation such as inhibition of 5′ cap formation, modulation of splicing, and blockade of polyadenylation of pre-mRNA in the nucleus [32] (Figure 1).

**Figure 2:**
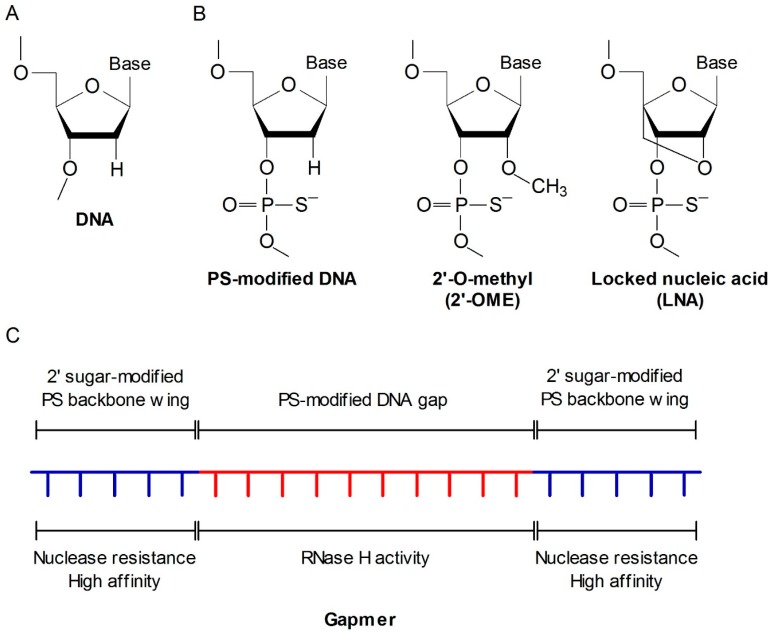
Chemical modifications of ASO. (**A**) Basic structure of a DNA oligonucleotide; (**B**) Chemical modifications of ASOs; (**C**) Structure of a gapmer. The 3rd generation of ASOs are mostly designed with a gapmer structure, typically with 20 nucleotides of phosphorothioate (PS)-modified backbone. The central sequence consists of approximately 10 simple PS-modified DNA residues, referred to as the gap, that can bind RNase H and induce the cleavage of target mRNA. Sugar-modified residues are overhanged on both ends of the gap as protective wings to resist nuclease activity and increase mRNA binding affinity.

As naked ASO can be easily degraded by nucleases in the biological fluid, various chemical modifications have been developed to improve its stability, potency, and safety in biological systems (Figure 2). Phosphorothioate (PS)-modified backbone is the most widely used chemistry in the first generation ASOs [33]. To further enhance stability in vivo and target mRNA binding affinity, second generation ASOs were designed with additional modifications such as 2′-*O*-methyl [2′-OME] or 2′-*O*-methoxyethyl [2′-MOE] coupled with PS backbone. However, 2′-sugar modifications block the recruitment of RNase H [34]. Therefore, a chimeric “gapmer” ASO structure was developed, which maintains a sequence of simple PS-modified backbone residues, referred to as the gap, to facilitate RNase H activity and sugar-modified residues on either side of the gap as protective wings [35]. The third generation ASOs were developed with a variety of modifications including peptide nucleic acid (PNA), locked nucleic acid (LNA), and phosphoroamide morpholino oligomer (PMO) in the ASO furanose ring, ribose sugar, and backbone structure, which further enhance their nuclear resistance, target affinity, and pharmacokinetics [36]. Several LNA-modified ASO drugs have already entered clinical trials [37].

Elevated levels of adenosine and increased expression of adenosine receptor A_1_ were detected in asthmatic patients. Inhaled adenosine binding to adenosine receptor A_1_ induced bronchoconstriction. Adenosine A_1_ receptor has been proposed as a target for asthma therapeutic intervention [38]. EPI-2010, a 21-mer PS-modified respirable ASO developed by Epigenesis Pharmaceuticals, was designed to target adenosine A_1_ receptor in the airways. Aerosolized EPI-2010 selectively reduced adenosine A_1_ receptor expression in vivo, which led to attenuated airway inflammation and bronchoconstriction, and increased surfactant level in experimental models of allergic asthma [39,40].

Mex-3B, muscle excess 3 RNA-binding family member B; BLT2, leukotriene B4 receptor 2; Ca(v)1, calcium voltage-gated channel subunit α1; VLA-4, very late antigen-4; NPRA, Natriuretic peptide receptor A; SOCS3, suppressors of cytokine signaling 3; Pdcd4, programmed cell death 4; RIP-2, receptor-interacting protein 2; PDE, phosphodiesterase; CHST3, carbohydrate sulfotransferase 3; PAI-1, plasminogen activator inhibitor 1; CTGF, connective tissue growth factor.IL-4, IL-5; and IL-13 are the priming cytokines for Th2 immune response and play a critical role in airway eosinophilia, AHR, and airway remodeling in asthma. In an OVA-sensitized murine airway inflammation model, an IL-4 ASO, complexed with polyethylenimine to improve intracellular delivery, was able to suppress IL-4 production up to 30% in the BAL fluid, decrease circulating lgE level, and alleviate lung inflammation [41]. Intravenous injection of a recombinant adeno-associated virus containing the IL-4 ASO (rAAV-asIL4) vector significantly suppressed airway inflammation, particularly eosinophilia in an OVA-challenged lung inflammation rat model [42]. In addition, rAAV-asIL4 ASO also ameliorated OVA-induced rat airway remodeling, characterized by a decrease in TGF-β1/TGF-β2-positive cells and a thickening of airway smooth muscle [43]. IL-5 is well known as a maturation and anti-apoptotic factor for eosinophils. Intravenous administration of an IL-5 ASO inhibited airway eosinophilia and antigen-induced late phase AHR, coincident with reduction of IL-5 protein levels in a murine OVA-challenged asthma model [44]. Similar to the strategy applied to the rAAV-asIL4 construction mentioned earlier, a viral vector containing IL-5 antisense (rAAV-ASIL-5) was transfected into allergic rats to investigate its effects on allergic inflammation and remodeling [45,46]. Expectedly, treatment with rAAV-ASIL-5 was found to inhibit BAL fluid and peripheral eosinophilia, serum lgE level, and BAL chemokine levels of eosinophilic cationic protein and eotaxin [45]. Application of rAAV-ASIL-5 was also able to decrease the numbers of TGF-β1/TGF-β2 positive cells in the peribronchial space and suppress the increase in total bronchial wall area and airway smooth muscle area [46].

IL-4 and IL-13 bind to their cognate receptors, both of which use the IL-4 receptor-α (IL-4Rα) subunit for full activation. Inhaled ASO targeting IL-4Rα showed the ability to reduce airway inflammation and attenuate airway hyperactivity in mouse asthma models [47,48]. In addition to IL-5, cytokine IL-3 and GM-CSF have also been shown to mediate airway eosinophilia and AHR. Simultaneous inhibition of IL-3, IL-5 and GM-CSF may benefit asthma control. Interestingly, receptors for IL-3, IL-5, and GM-CSF use the common β chain (βc) as their receptor subunit, and ASO-mediated gene silencing of βc can be a promising strategy. Several chemokines, especially eotaxin, help to attract eosinophilia into the airway via CC chemokine receptor 3 (CCR3) in asthma. Inhibition of GM-CSF/IL-3/IL-5 signaling by ASOs targeting the βc of their receptors or CCR3 expression in vivo suppressed antigen-induced eosinophilia and AHR in rat models of allergic asthma [49,50]. It has been shown that combining ASOs targeting βc and CCR3 simultaneously was superior in preventing antigen-induced eosinophilia and AHR, rather than employing ASOs against either target alone [51].

Anti-tumor necrosis factor (TNF)-α therapeutics have the potential to alleviate allergic inflammation. Local administration of TNF-α ASO remarkably inhibited TNF-α expression decreased inflammatory cell infiltration and blocked mucus hypersecretion, which was associated with increased CD4+CD25+FoxP3+ regulatory T cells and reduced Th2 cells in an OVA-induced murine asthma model [52]. In recent years, IL-33 has been recognized as a key pro-inflammatory cytokine that mediates allergic airway inflammation. Muscle excess of 3 RNA-binding family member B (Mex-3B) could directly upregulate IL-33 expression by inhibiting miR-487b-3p-mediated repression of IL-33. Inhalation of an ASO targeting Mex-3B suppressed airway eosinophilia, lung inflammation, mucus hypersecretion, and BAL fluid levels of Th2 cytokine IL-4, IL-5 and IL-13 in an experimental model of asthma [53].

The B7-family molecule CD86, expressed on the surface of antigen-presenting cells in the lungs, delivers essential costimulatory signals to prime naive T-cells upon allergen activation. Inhaled CD86 ASO suppressed pulmonary inflammation and AHR in allergic mice [54]. Transcription factor GATA binding protein 3 (GATA3) and signal transducer and activator of transcription 6 (STAT6) are essential Th2-driven transcription factors. Gene silencing of GATA3 or STAT6 expression by ASO was found to abrogate Th2-mediated airway inflammation in murine asthma models [55,56]. NF-κB is another essential transcription factor driving inflammatory diseases, including asthma. Intravenous administration of NF-κB subunit p65 ASO, either prophylactically or therapeutically, was able to inhibit all the asthmatic symptoms, including BAL fluid eosinophilia, elevation of Th2 cytokines, serum lgE level, and AHR in a mouse asthma model [57]. Intracellular kinases are important signaling mediators that transduce cellular responses, including inflammatory signals. Activation of the protein tyrosine kinase Syk, following cross-linking of Fc receptors, led to the release of pro-inflammatory mediators during airway inflammation. The mitogen-activated protein kinase (MAPK) p38 pathway was reported to be critically important for the activation of the IL-5 receptor and eotaxin receptors in eosinophils, resulting in survival, activation, degranulation, and chemotaxis of eosinophils in the airway. Respirable ASO targeting protein tyrosine kinase Syk or MAPK p38α, markedly inhibited OVA-induced lung tissue eosinophilia, airway mucus hypersecretion, and AHR in rodents [58,59]. Lipid mediators such as leukotrienes (LTs) play an important role in the pathogenesis of asthma. BLT2 is a low-affinity receptor for LTB4, a potent lipid mediator of inflammation generated from arachidonic acid. Intravenous administration of an ASO targeting BLT2 markedly attenuated airway inflammation and AHR in vivo, probably through the blockade of reactive oxygen species and the subsequent activation of NF-κB [60].

Dihydropyridines selectively bind to voltage-dependent calcium channels in activated Th2 cells to modulate the cell function. Intranasal delivery of calcium voltage-gated channel subunit α1 (Ca(v)1) ASO suppressed airway inflammation and hyperreactivity in an OVA-challenged murine asthma model. In a passive asthma model, where OVA-specific Th2 cells transfected with Ca(v)1 ASO were adoptively transferred into naive mice followed by challenged with OVA, it was shown that Th2 cells transfected with Ca(v)1 ASO had impaired Ca(2+) signaling and Th2 cytokine production and had also lost their ability to induce airway inflammation in vivo [61]. Adhesion molecule very late antigen (VLA)-4 is an α_4_β_1_ integrin localized on many inflammatory cells, which plays an important role in cell adhesion, trafficking, and activation. VLA-4 has been implicated in the recruitment of eosinophils and T cells. Aerosol delivery of VLA-4 ASO reduced eosinophil recruitment, mucus secretion, and AHR in an experimental asthma model [62]. ATL1102, developed by Antisense Therapeutics, is a second generation ASO targeting CD49d, a subunit of VLA-4. To our knowledge, this drug showed efficacy in patients with relapsing-remitting multiple sclerosis in a Phase II clinical trial and has been licensed for clinical trial in asthma patients [63].

PDEs are promising therapeutic targets for chronic inflammatory diseases. Roflumilast, a selective PDE4 inhibitor, has been approved for the treatment of COPD [16]. It has been reported that combined PDE4 and PDE7 inhibition are more effective at controlling inflammation [64,65]. In a cigarette smoke-induced lung inflammation model, combined respirable PDE4B/4D and PDE7A ASOs exerted more potent and broader anti-inflammatory actions against cigarette smoke-induced lung inflammation than oral roflumilast [66].

A number of ASOs targeting different signaling pathways involved in pathogenesis of pulmonary fibrosis have also been investigated. Other than TGF-β, basic fibroblast growth factor (bFGF) is also a potent mitogen for fibroblast proliferation and differentiation, which may contribute to pulmonary fibrosis development. Respirable bFGF ASO showed promise in preventing bleomycin-induced pulmonary fibrosis, likely through attenuating bFGF-mediated activation of the TGF-β1/Smad signaling mechanism in a rat model [67]. TNF-α has also been associated with pro-fibrotic activity. Local administration of TNF-α ASO demonstrated an anti-fibrotic effect, probably via a sustained increase of regulatory T cells, which inhibited pro-fibrotic mediator production and extracellular matrix deposition in a bleomycin-induced pulmonary fibrosis mouse model [68]. Pro-inflammatory transcription factor NF-κB p65 subunit has also been shown to play an essential role in the development of pulmonary fibrosis. Interruption of NF-κB transcription activity by p65 ASO attenuated bleomycin-induced fibrosis and the expression of α-SMA in a mouse fibrosis model [69].

EPI-2010, the A_1_ adenosine receptor ASO, had progressed to clinical trial and was found to be safe and well-tolerated in human subjects [70]. While it demonstrated modest efficacy in patients with mild asthma, EPI-2010 failed to produce sufficient additional benefit in moderate asthmatics after ICS therapy. Product development was discontinued after the Phase II trial [40,70]. TPI ASM8, developed by Topigen Pharmaceuticals, is an ASO drug that consists of two PS-modified ASOs, one targeting the common βc chain of IL-3, IL-5, and GM-CSF receptors, and the other targeting chemokine receptor CCR3. TPI ASM8 demonstrated safety and well-toleration in human trials. In Phase II trials, inhaled TPI ASM8 in mild asthmatics reduced sputum eosinophil influx, both early and late asthmatic responses after allergen challenge [71,72,73]. Although TPI ASM8 is currently not in active trials, it is the view of these authors that it is promising for the treatment of moderate to severe asthma. AIR645, developed by Altair Therapeutics, is another ASO drug that is designed to target IL-4α for both Th2 cytokine IL-4 and IL-13. It was demonstrated that nebulized AIR645 was well-tolerated in healthy volunteers in a Phase I trial, but failed to show enough efficacy in asthmatic patients in a Phase II trial [74]. Thus, further development of AIR645 for asthma treatment has been halted. TPI 1100 is another dual ASO drug developed by Topigen Pharmaceuticals, targeting both PDE4B/4D and 7A isoforms for the treatment of COPD. A Phase I clinical trial certificate was approved for testing in healthy subjects, but the trial was withdrawn prior to enrollment. Table 1 summarizes the five ASO drugs for the treatment asthma or COPD in clinical trials.

### 2.2. siRNA

RNA interference (RNAi) is a natural biological process in which double-strand RNA molecules induce sequence-specific reduction of target mRNAs [75]. By taking advantage of this target gene silencing process, exogenous siRNA is widely used in interpreting gene function on a genome-wide scale [76]. siRNA is a functional unit of the RNAi pathway [77]. siRNA is a double-stranded RNA of 21 to 23 nucleotides, consisting of an antisense or guide strand and a sense or passenger strand [78,79,80].

**Figure 3:**
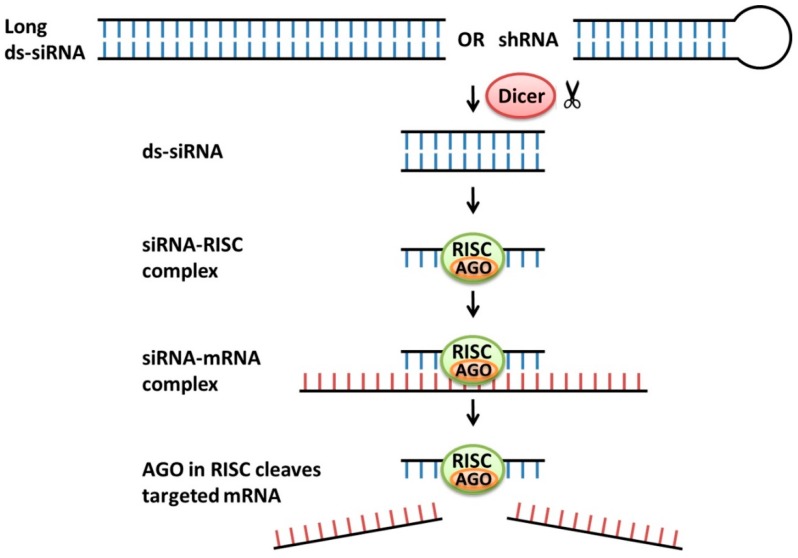
Mechanisms of actions of siRNAs**.** Long double-stranded RNA (Long ds-RNA) or short hairpin RNA (shRNA) is processed and cleaved into a 20–30 bp double-stranded siRNA (ds-RNA) by RNase Dicer. In cell cytoplasm, the antisense strand of an siRNA binds to the endogenous RNA-induced silencing complex (RISC), whereas the sense strand of the siRNA is discarded. The antisense strand guides RISC to recognize target mRNA through base-pair complementary binding. The endonuclease Argonaute 2 (AGO) in the RISC complex enzymatically cleaves the target mRNA, leading to target gene knockdown.

Chemically synthesized siRNAs can be either transfected into a cellular system directly as short double-stranded RNAs (dsRNAs), or introduced as long dsRNAs or short hairpin RNAs (shRNAs), which are subsequently processed to siRNAs by the RNase III family protein Dicer [78,79]. The antisense strand of the short dsRNA binds to the endogenous cytoplasmic RNA-induced silencing complex (RISC) [81] and guides the RISC to recognize target mRNA through base-pair complementary binding [79]. Argonaute 2 enzymatically cleaves the target mRNA in the RISC complex, leading to target gene knockdown [82]. The cleavage products are released and degraded, and the siRNA-RISC complex is free to bind to another mRNA target [83]. Figure 3 briefly summarizes the mechanism of gene knock down by siRNAs.

Many strategic targets implicated in the pathogenesis of asthma have been investigated via loss-of-function studies using siRNA. IL-4, IL-5, and IL-13 are essential Th2 cytokines in driving the development of asthma. siRNAs targeting IL-4 and respiratory syncytial virus (RSV) simultaneously were studied in a mouse model of virus-induced asthma exacerbation. Combined anti-IL-4 and anti-RSV siRNAs were able to significantly reduce total cell count and eosinophilia in BAL fluid, downregulate both IL-4 and RSV viral RNA expression, and attenuate AHR, but upregulate IFN-γ level in lung tissues [84]. A lentivirus-delivered IL-5 siRNA was found to moderate cell infiltration as well as AHR in a mouse asthma model [85]. Natriuretic peptide receptor A (NPRA) is the primary receptor for atrial natriuretic peptide (ANP), which has been associated with allergic inflammation and asthma. Allergic inflammation was found attenuated in mice deficient in NPRA [86]. Interestingly, transdermal administration of NPRA siRNA nanoparticles significantly reduced eosinophilia, lung histopathology, pro-inflammatory cytokines IL-4 and IL-5, and AHR in an OVA-induced mouse asthma model [87]. In line with the gene silencing effect by STAT6 ASO [56], the knocking down of STAT6 by siRNA also inhibited allergic airway inflammation and AHR in mice [88]. CD86 is another target which was firstly tested using ASO knockdown in an asthma model. Gene silencing of CD86 by siRNA also decreased airway eosinophilia, BAL fluids IL-5 and IL-13, and CCL17 production, serum OVA-specific IgE levels, and AHR in an OVA-induced mouse asthma model [89]. Suppressors of cytokine signaling (SOCS) is a family of proteins that modulate cytokine signaling as negative regulators. SOCS3 is predominantly expressed in Th2 cells and selectively inhibits Th1 cytokine IL-12 signaling and T-bet expression in these cells, which favor the development of Th2-mediated allergic response [90]. SOCS3 has been proposed as a therapeutic target for asthma. Knockdown of SOCS3 by siRNA reduced airway eosinophilia, ameliorated AHR, and improved airway remodeling via the inhibition of phosphorylation of STAT3 and the suppression of the RhoA/Rho kinase pathway in an OVA-induced chronic asthma model [91]. Programmed cell death 4 (Pdcd4), initially known as a tumor-suppressor gene, has also been associated with severe asthma in children [92]. In an OVA-induced rat asthma model, Pdcd4 was found up-regulated in rat lung tissue and gene silencing of Pdcd4 by siRNA reduced inflammatory cell infiltration and ameliorated airway remodeling [93]. c-kit, best known as a proto-oncogene, is a receptor tyrosine kinase for cytokine stem cell factor (SCF). Numerous studies have indicated that c-kit signaling pathway plays an important role in the development of allergic inflammation [94]. Intranasal siRNA nanoparticles targeting c-kit reduced infiltration of eosinophils in BAL fluid, downregulated the production of SCF, IL-4, and IL-5, and suppressed airway mucus secretion in an OVA-induced mouse asthma model [95]. In our laboratory, we showed that gene silencing of receptor-interacting protein (RIP)-2, a positive NF-κB regulator by siRNA, abated inflammatory cell infiltration, mucus hypersecretion, and AHR in mice [96].

Carbohydrate sulfotransferase 3 (CHST3) is an enzyme catalyzing the sulfation of chondroitin leading to the formation of carbohydrate sulfate proteoglycans, important mediators for leukocyte trafficking and inflammation. CHST3 was found elevated in an elastase-induced murine model of pulmonary emphysema. Intraperitoneal administration of siRNA targeting CHST3 attenuated lung inflammation, restored the elastin level, and improved lung function and emphysema [97]. This study suggested that RNA interference of CHST3 may have a therapeutic value for COPD.

TGF-β1 is the essential mediator in the pathogenesis of pulmonary fibrosis. Aerosolized siRNA targeting TGF-β1 mRNA significantly inhibited bleomycin-induced pulmonary fibrosis in both acute and chronic mouse models in a dose-dependent manner. In addition, aerosolized human TGF-β1-specific siRNA also efficiently inhibited pulmonary fibrosis, improved lung function, and prolonged survival in a novel spontaneous pulmonary fibrosis model developed in mice overexpressing the full length of human TGF-β1 in the lungs [98]. Plasminogen activator inhibitor-1 (PAI-1) blocks the fibrinolytic activity of urokinase-type plasminogen activator. In bleomycin-induced pulmonary fibrosis mouse and rat models, the suppression of PAI-1 by siRNA significantly reduced the deposition of collagen and attenuated pulmonary fibrosis [99,100]. Connective tissue growth factor (CTGF) regulates fibroblast mitosis and promotes collagen deposition in pulmonary fibrosis. CTGF siRNA was able to ameliorate collagen deposition, inflammatory cytokines production, and pulmonary fibrosis in a bleomycin-induced rat model [101].

The only siRNA that reached the clinical trial stage for respiratory disease targets Syk kinase, activation of which leads to release of pro-inflammatory mediators during airway inflammation (Table 1). As discussed earlier, aerosolized Syk ASO was able to suppress many of the essential mediators of OVA-induced allergic inflammation in a rat asthma model [58]. Gene silencing of Syk using siRNA in bronchial epithelial cells showed decreased levels of inflammatory mediators including IL-6, ICAM-1, and NO [102,103,104]. While the effect of Syk RNA interference was only demonstrated in an asthma model using ASO, but not siRNA, an inhaled siRNA drug candidate named Excellair^TM^, which was designed to silence Syk (ZaBeCor Pharmaceuticals, Bala Cynwyd, PA, USA), entered a Phase II clinical trial in asthma patients in 2009 following positive Phase I trial results, but the Phase II trial was discontinued in 2015. It remains presently unknown if Excellair^TM^ exhibited any clinical activity.

### 2.3. miRNA

**Figure 4:**
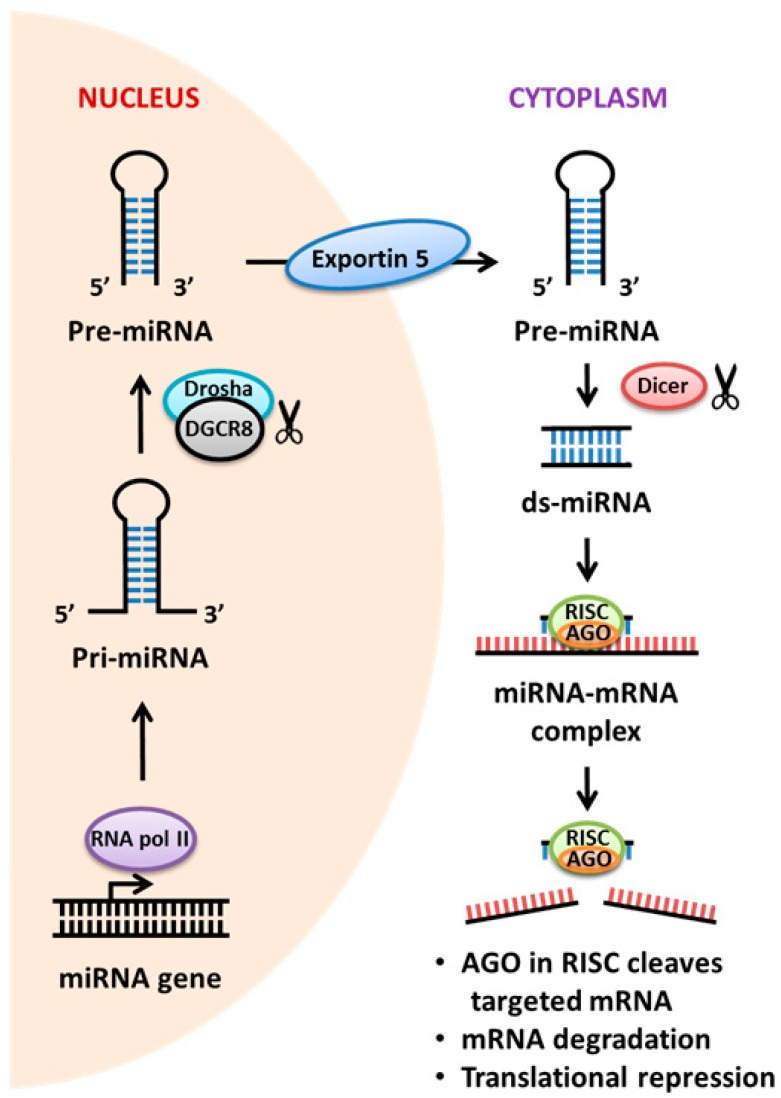
Mechanisms of actions of miRNAs. The miRNA gene is transcribed into a primary miRNA trancript (pri-miRNA), which is further excised into pre-miRNA by RNase Drosha. The pre-miRNA is exported to the cytoplasm by Exportin 5 and spliced by RNase Dicer to produce a double-stranded miRNA (ds-miRNA) duplex. A single-stranded mature miRNA is generated from the miRNA duplex by helicases and assembled into a RISC complex. The endonuclease Argonaute 2 (AGO) in the RISC complex enzymatically cleaves the target mRNA, leading to target gene knockdown, typically on a base-pair complementary basis. In addition, miRNA also binds mRNA coding regions and intron-exon junctions to modulate the target gene expression levels by either mRNA degradation or protein translation repression.

It has been observed that only 2% of the human genome is transcribed into protein products, but the majority of the human genome is converted to non-coding RNA (ncRNAs) [105]. MicroRNAs (miRNAs) are small endogenous ncRNAs, which function to regulate target gene expression transcriptionally and post-transcriptionally [106]. Initially, it was thought that miRNA has no biological importance. However, it has recently been shown to play fundamental roles in proliferation, differentiation, apoptosis, and inflammation [107,108]. To date, over 2500 mature miRNAs have been identified in the human genome, but many of their biological functions remain to be uncovered [109]. In addition, it is estimated that the expression of more than 60% of human protein-coding genes may be regulated by miRNAs [110]. miRNAs are primarily encoded either within the introns of protein-coding genes or inter-genic regions of genomic DNA [106]. The biogenesis of miRNAs involves a series of well-orchestrated but complex steps, and a schematic illustrating the mechanism of miRNA processing and function is demonstrated in Figure 4. A primary transcript (pri-miRNA) with a length of several hundred to several thousand nucleotides is first transcribed by RNA polymerase II within the nucleus [106,111]. The pri-miRNA is subsequently cleaved by the nuclear microprocessor complex, consisting of the RNase III enzyme, Drosha, and the doubled stranded RNA-binding protein DiGeorge syndrome critical region gene 8 (DGCR8), into a hairpin precursor miRNA (pre-miRNA) of approximately 70 nucleotides [112]. The resulting pre-miRNA is exported to the cytoplasm by Exportin 5 before it undergoes processing by the cytoplasmic RNase III enzyme, Dicer. The double-stranded miRNA duplex, generated upon cleavage by the Dicer, is approximately 22 nucleotides long and is unwound by helicases [113,114,115]. Argonaute 2 protein then facilitates the intercalation of the mature miRNA strand into the multi-protein complex RISC, where the RISC complex will typically guide the miRNA to use its highly specific “seed sequence” at 5’end to selectively bind to the miRNA recognition elements within the 3’-untranslated region (UTR) of target mRNA [116,117,118]. Apart from binding within the 3’-UTR of mRNA transcripts, miRNA also binds the 5′-UTR mRNA regions, mRNA coding regions, and intron-exon junctions to modulate the target gene expression levels by either protein translation inhibition or RNA degradation [108,119,120,121]. Moreover, miRNAs can also be secreted from cells into circulation in a form of exosomes, which enable cellular communication in an autocrine or paracrine manner [122,123,124]. Several studies have also shown that miRNA is pivotal for both innate and adaptive immune responses to protect against invading foreign harmful particles [119,125].

Through miRNA profiling on samples from murine models and diseased patients, there is growing documentation to implicate miRNAs in the development of respiratory diseases. miRNAs have the potential to be used as biomarkers for disease diagnosis and as therapeutic targets for disease management.

The assessment of miRNA expression in human airway-infiltrating T cells from asthmatics identified increased levels of miR-19a, which have been described to be involved in the up-regulation of Th2 cytokine production and the amplification of various inflammatory signaling pathways, including JAK-STAT and NF-κB pathways [126]. In an asthma exacerbation model, exposure of combined IFN-γ and LPS to mice synergistically increased miR-9 expression and induced steroid-resistant AHR [127]. Inhibition of miR-9 with sequence-specific antagomir promoted both protein phosphatase 2A activity and dexamethasone-induced glucocorticoid receptor nuclear translocation and restored steroid sensitivity to AHR [127]. Additionally, knockdown of miR-106a by antagomir was demonstrated to increase lung IL-10 expression as well as to suppress asthmatic phenotypes including airway inflammation, goblet cell metaplasia, subepithelial fibrosis, and AHR [128,129]. In miR-155 knockout mice, the deficiency of miR-155 decreased the severity of asthma via the alteration of Th2 cell function [130,131]. MiR-221 was also reported to be differentially expressed between the asthmatics and the controls, and blockade of miR-221 using anti-miR-221 oligonucleotide mitigated airway inflammation in a mouse asthma model [132]. In the human lung epithelial cell line A549, a high concentration of pro-inflammatory IL-β induced an increase in miR-146a and, to a lesser extent, miR-146b expression. Further study demonstrated that IL-1β-induced miR-146a, but not miR-146b, negatively regulated IL-β-induced chemokines IL-8 and RANTES release via the NF-κB and JNK-1/2 pathways in these cells. These studies indicated that a rapid increase in miRNA-146a expression could provide a novel mechanism for the negative regulation of severe inflammation during the innate immune response [133,134]. In human airway smooth muscle cells, miR-146a and miR-146b expressions were found more inducible in the cells isolated from asthmatic subjects than those from normal subjects upon stimulation with cytomix (IL-1β, TNF-α, and IFN-γ). Transfection of either miR-146a or miR-146b mimics in smooth muscle cells reduced inflammatory mediator cyclooxygenase-2 and IL-1β expression, whereas loss-of-function studies using miR-146a and miR-146b inhibitors showed that miR-146a, but not miR-146b, was an endogenous negative regulator to suppress cyclooxygenase-2 and IL-1β expression in these cells. These results suggested miR-146 mimics may be promising candidates as anti-inflammatory treatments for asthma [135].

Studies have reported that multiple miRNAs were altered in COPD patients and in vivo murine models. Among the various miRNA profiling studies using a qRT-PCR array, several have validated that a number of circulating miRNAs could serve as potential biomarkers for the detection and prognosis of COPD; down-regulation of miR-20a, 28-3p, 34c-5p, 100, and 181a, and up-regulation of miR-7 and 21 [136,137]. The levels of serum miR-21 and 181a and their ratios might be used for the evaluation and prediction of COPD development in asymptomatic heavy smokers [137]. The expression of miR-199a-5p was found to be downregulated in peripheral blood monocytes and regulatory T cells from COPD patients, which might be responsible for a significant increase in expression of numerous unfolded protein response markers and cytokines [138,139]. In the lungs of cigarette smoke-exposed mice, activation of IL-1R1 mediated the elevation of miR-135b, which then suppressed the endogenous IL-1R1 expression, suggesting a negative regulatory feedback mechanism by miR-135b in resolving cigarette smoke-induced inflammation [140].

Evidence for the involvement of miRNA in the pathogenesis of IPF has been emerging recently. MiR-26a was found to be downregulated in a mouse pulmonary fibrosis model, which resulted in increased levels of connective tissue growth factor, enhanced collagen deposition, and lung fibrosis [141]. A separate study found that decreased miR-200 expression promoted fibrogenic activity of pulmonary fibroblasts in a mouse fibrosis model, and restoration of miR-200 was found to reverse the fibrosis process by inhibiting TGF-β1-induced epithelial-mesenchymal transition [142]. MiR-326, capable of regulating pro-fibrotic TGF-β1, was identified to be down-regulated in mouse fibrotic lungs as well as in human IPF lungs [143]. MiR-485-5p was another miRNA found to be reduced in the lung tissues of IPF patients and its overexpression could abrogate the pulmonary fibrosis development in a murine model [144]. Table 2 summarizes ASO, siRNA and miRNA therapeutic targets or biomarkers for asthma, COPD, and pulmonary fibrosis.

Even though no clinical trials have been performed to assess the impacts of miRNA-targeted therapies in respiratory diseases, two human clinical trials that focus on miRNA as directed therapeutics in other human diseases are currently ongoing. The first miRNA clinical trial on miravirsen, an anti-miR-122 therapy against hepatitis C virus infection, was completed by Santaris Pharma with some positive results in a Phase II trial, but there appeared to be no plans for advancing the drug into a Phase III trial [145]. The other one using a miR-34 mimic, MRX34, for the treatment of advanced hepatocellular carcinoma was terminated recently in a Phase I clinical trial due to immune related serious adverse events [145,146]. Taken together, these findings raised the possibility of using miRNA molecules as novel therapeutic targets for human lung diseases in the near future. Several approaches can be employed to modulate pathological miRNA dysregulation, which range from the use of anti-miRs oligonucleotides/antagomirs or miRNA sponges, to inhibiting upregulated miRNA, to the pharmacological activation of miRNA function by administrating double-stranded synthetic miRNA oligonucleotide mimics into target cells [108,147]. However, while the targeting of single miRNA is beneficial as it can have broader effects on multiple genes and cellular pathways than can siRNA [148], the translation of miRNA biology to clinical application in respiratory disease remains challenging.

## 3. Future Perspective

Since the first report describing the use of ASO targeting Rous sarcoma virus 35S RNA as a potential oligonucleotide therapy, there has been an exponential increase in the discovery and development of oligonucleotides as a therapeutic modality [30]. ASO, siRNA, and miRNA are the three major types of oligonucleotide therapeutics under development. There are a few advantages for targeting RNA over protein molecules. As a single copy of RNA could be a template for the synthesis of multiple copies of a protein, it would be more efficient to regulate the mRNA level rather than the protein level to block the protein function [149]. Another benefit of using oligonucleotide strategy comes from the complete decoding of human genome, which greatly shortens the time between identification of gene targets and the design and validation of oligonucleotide products. Finally, several different oligonucleotide sequences can be combined into one single drug product, which is especially important for diseases with multifactorial etiology. Aside from ASO, siRNA, and miRNA, another recently described RNA class, long non-coding RNA (lncRNA), has also been implicated in respiratory diseases including asthma, COPD, and fibrosis [150,151,152]. A number of promising therapeutic strategies to regulate lncRNA are in the pre-clinical stage, even though big challenges and obstacles are expected [153]. One big challenge of lncRNAs for therapeutic development is their poor conservation between species. So even if one lncRNA can be demonstrated as a successful therapeutic target in pre-clinical animal models, it might not be translated into humans. Thus, it seems more important to investigate human lncRNAs directly for therapeutic purposes [153].

Chronic respiratory diseases are uniquely suitable for the inhalation route of administration. In the lungs, uptake of aerosolized oligonucleotides has been demonstrated in immune cells like alveolar macrophages, as well as in structural cells such as bronchial and alveolar epithelial cells [154,155]. Absorption of oligonucleotides by inhalation, occurring mainly within the respiratory tracts with minimum systemic absorption, is a proposed advantage over oral or systemic administration. However, two industry-originating reports revealed that the bulk dose of PS-modified and PS-LNA-modified oligonucleotides could accumulate systemically in a biologically active fashion in rodents subjected to intratrachael administration [156,157]. Nevertheless, the effective and uniform intracellular delivery of oligonucleotides to their site of action still remains a challenge. Oligonucleotide delivery strategies commonly used in in vitro cell culture models such as cationic lipids, polymers, and electroporation are not suitable for in vivo application due to large particle size, pharmacokinetics constraints, and toxicity [158,159]. To date, most inhaled oligonucleotide products that have demonstrated efficacy in humans through aerosolization of simple aqueous solutions do not contain any carriers [160]. To improve in vivo delivery and efficacy, new strategies like ligand-oligonucleotide conjugates, antibody-targeted conjugates, and engineered viral vector-, lipid- and polymer-based nanoparticles may be further evaluated and developed [27,161].

Several shortcomings arising from the intrinsic properties of oligonucleotides may challenge oligonucleotide therapies. These include their stability in vivo, associated non-specific immune responses, and off-target cytotoxicity. dsRNA can activate innate immune responses and increase interferon production through recognition of Toll-like receptors (TLR)3, TLR7, and retinoic acid-inducible gene I protein. Specific chemical modification, such as 2′-OME modification, can largely block binding and activation of these receptors and reduce immune responses. In addition, partial sequence complementarity of mRNAs with oligonucleotides may lead to sequence-specific off-target effects, which can be minimized by chemical modification, alternative sequence selection, and the use of pools of oligonucleotides that dilute off-target effects by reducing the concentration of individual oligonucleotide [78,162]. Although the completed Phase I and Phase II studies with EPI-2010, TPI ASM8, and AIR645 have not shown any major side effects thus far, safety studies with longer duration will need to be monitored closely as asthma, COPD, and fibrosis are chronic disorders that require long-term drug management.

Although recently there have been a few monoclonal antibodies approved by the FDA for the treatment of asthma, namely omalizumab, mepolizumab, and reslizumab, the only viable route of administration for these antibodies is via injection, which is considered invasive [163,164,165]. Oligonucleotides, on the other hand, can be given locally into the airways in a less invasive way, such as through aerosolized inhalation. Although topically-administrated dsRNA could trigger non-specific innate immune responses in the airway; systemically-delivered monoclonal antibodies may produce much more pronounced life-threatening adverse effect such as a hypersensitivity reaction [166]. Another advantage of oligonucleotide over monoclonal antibody therapy is that oligonucleotide can theoretically target any molecule as it can penetrate cells, whereas a monoclonal antibody can mainly target extracellular or cell surface molecules. Nevertheless, monoclonal antibody and oligonucleotide strategies are two different treatment modalities with their own pros and cons.

In summary, oligonucleotide therapeutics is a promising drug modality and is especially ideal for respiratory diseases due to its convenient topical airway administration and minimum systemic toxicity. However, substantial resources are required for mechanistic studies and pre-clinical model verification to further enhance delivery, increase efficacy, and reduce toxicity for this novel therapeutic approach. Nevertheless, oligonucleotide drugs are holding promise to be translated from bench to bedside and to become the major thrust for new pharmaceuticals in the next decade.

## Figures and Tables

**Table 1:** Oligonucleotide drugs for asthma, COPD and pulmonary fibrosis entered into clinical trials.

Drug Name	Type	Target	Disease	Company	Status
EPI-2010	ASO	Adenosine A1 Receptor	Asthma	Epigenesis	Phase II/Discontinued
TPI ASM8	ASO	Combined βc & CCR3	Asthma	Topigen	Phase II
AIR645	ASO	IL-4/IL-13 Receptor α chain	Asthma	Altair/ISIS	Phase II/Discontinued
TPI 1100	ASO	Combined PDE4B/4D/7A	COPD	Topigen	Phase I/Discontinued
Excellair	siRNA	Syk Kinase	Asthma	ZaBeCor	Phase II/Discontinued

**Table 2:** Oligonucleotide therapeutic targets or biomarkers for asthma, COPD, and pulmonary fibrosis.

Therapeutic Targets or Biomarkers						
Disease	ASO	Ref.	siRNA	Ref.	miRNA	Ref.
Asthma	Adenosine A_1_ Receptor	[39,40]	IL-4	[84]	miR-19a ^#^	[126]
	IL-4	[41,42,43]	IL-5	[85]	miR-9	[127]
	IL-5	[44,45,46]	NPRA	[87]	miR-106a	[128,129]
	IL-4α	[47,48]	STAT6	[88]	miR-155	[130,131]
	βc & CCR3	[49,50,51]	CD86	[89]	miR-221	[132]
	TNF-α	[52]	SOCS3	[91]	miR-146	[133,134,135]
	Mex-3B	[53]	Pdcd4	[93]		
	CD86	[54]	c-kit	[95]		
	GATA-3	[55]	RIP-2	[96]		
	STAT6	[56]				
	NF-κB p65 Subunit	[57]				
	Syk Kinase	[58]				
	p38α MAPK	[59]				
	BLT2	[60]				
	Ca(v)1	[61]				
	VLA-4	[62]				
COPD	PDE4B/4D/7A	[66]	CHST3	[97]	miR-20a ^#^, -28-3p ^#^	[136]
					miR-34c-5p ^#^, -100 ^#^, -7 ^#^	[136]
					miR-21 ^#^, -181a ^#^	[137]
					miR-199a-5p ^#^	[138,139]
					miR-135b	[140]
IPF	bFGF	[67]	TGF-β1	[98]	miR-26a ^#^	[141]
	TNF-α	[68]	PAI-1	[99,100]	miR-200	[142]
	NF-κB p65 Subunit	[69]	CTGF	[101]	miR-326 ^#^	[143]
					miR-485-5p	[144]

^#^ Biomarker. Mex-3B, muscle excess 3 RNA-binding family member B; BLT2, leukotriene B4 receptor 2; Ca(v)1, calcium voltage-gated channel subunit α1; VLA-4, very late antigen-4; NPRA, Natriuretic peptide receptor A; SOCS3, suppressors of cytokine signaling 3; Pdcd4, programmed cell death 4; RIP-2, receptor-interacting protein 2; PDE, phosphodiesterase; CHST3, carbohydrate sulfotransferase 3; PAI-1, plasminogen activator inhibitor 1; CTGF, connective tissue growth factor.

## Abbreviations

AHR	airway hyperresponsiveness
Ago	Argonaute 2
ASO	antisense oligonucleotide
BAL	bronchoalveolar lavage
CCR	CC chemokine receptor
CHST	carbohydrate sulfotransferase
COPD	chronic obstructive pulmonary disease
CTGF	connective tissue growth factor
DGCR	DiGeorge syndrome critical region gene
ds	double-stranded
FGF	fibroblast growth factor
ICS	inhaled corticosteroid
IPF	idiopathic pulmonary fibrosis
LNA	locked nucleic acid
lncRNA	long non-coding RNA
LT	leukotriene
MAPK	mitogen-activated protein kinase
miRNA	microRNA
ncRNA	non-coding RNA
NPRA	natriuretic peptide receptor A
OVA	ovalbumin
PAI	plasminogen activator
PDE	phosphodiesterase
PMO	phosphoroamide morpholino oligomer
PNA	peptide nucleic acid
PS	phosphorothioate
RISC	RNA-induced silencing complex
RNAi	RNA interference
RSV	respiratory syncytial virus
SCF	stem cell factor
sh	short hairpin
siRNA	small interfering RNA
SOCS	suppressor of cytokine signaling
STAT	signal transducer and activator of transcription
TGF	transforming growth factor
TLR	Toll-like receptor
TNF	tumor necrosis factor
UTR	untranslated region
VLA	very late antigen
